# Protective Effects of *Momordica charantia* Extract on Dexamethasone-Induced Sarcopenic Changes in C2C12 Cells: Integrated Network Pharmacology and Experimental Validation

**DOI:** 10.3390/ph19060893

**Published:** 2026-06-04

**Authors:** Jung Eun Park, Kang Sub Kim, Mina Jeong, Hee Woon Ann, Rajath Ramachandran, Il-Ho Park, Ki Hyun Kim, Ki Sung Kang, Dae-Woon Eom

**Affiliations:** 1 College of Korean Medicine, Gachon University, Seongnam 13120, Republic of Korea; ppp1416@gachon.ac.kr (J.E.P.); kagnsub@gachon.ac.kr (K.S.K.); jmina@gachon.ac.kr (M.J.); heewoon05@naver.com (H.W.A.); rajath@gachon.ac.kr (R.R.); 2 College of Pharmacy, Sahmyook University, Seoul 01795, Republic of Korea; parkilho@syu.ac.kr; 3 School of Pharmacy, Sungkyunkwan University, Suwon 16419, Republic of Korea; 4 Department of Pathology, University of Ulsan College of Medicine, Gangneung Asan Hospital, Gangneung 25440, Republic of Korea

**Keywords:** *Momordica charantia*, sarcopenia, myogenesis, p38 MAPK, network pharmacology

## Abstract

**Background/Objectives**: Sarcopenia is characterized by progressive skeletal muscle loss and impaired myogenic differentiation and is closely associated with inflammation and metabolic dysfunction. **Methods**: This study investigated the protective effects of *Momordica charantia* extract against dexamethasone-induced sarcopenia and explored the underlying mechanisms using network pharmacology, C2C12 cell-based assays, Western blotting, and molecular docking. Network pharmacology analysis identified quercetin, ascorbic acid, and tocopherol as major active compounds associated with targets related to inflammation, extracellular remodeling, and metabolic dysfunction. **Results**: *M. charantia* extract (MCE) did not markedly reduce cell viability at concentrations up to 100 μg/mL and improved dexamethasone-induced morphological impairment of myotubes. The extract reduced MAFbx, MMP-2, and MMP-9 expression while restoring phosphorylated p38, MyoD, and myogenin expression, indicating suppression of atrophy- and remodeling-related responses, together with the recovery of myogenic signaling. Among the major identified compounds, all attenuated dexamethasone-induced myotube atrophy and quercetin showed the most pronounced morphological recovery. Molecular docking analysis targeting p38α showed the highest binding affinity for α-tocopherol, followed by quercetin and ascorbic acid, supporting potential interactions between the major compounds and p38 MAPK-related signaling. **Conclusions**: Collectively, these findings suggest that *M. charantia* attenuates sarcopenic changes by promoting myogenic differentiation and modulating the p38 MAPK-associated pathways.

## 1. Introduction

Sarcopenia is a progressive disorder characterized by the loss of skeletal muscle mass and function, and has emerged as a major public health concern in aging societies [1]. Beyond normal aging, it is closely associated with physical dysfunction, increased risk of falls, reduced quality of life, and high mortality, particularly in relation to metabolic diseases such as diabetes [2]. Skeletal muscle is not only a motor organ but also a critical metabolic engine, accounting for approximately 80% of insulin-mediated glucose uptake. In patients with type 2 diabetes mellitus (T2DM), metabolic homeostasis is disrupted by a constellation of shared pathological features that are also observed in sarcopenia [3,4]. Although traditionally viewed as distinct conditions, recent epidemiological evidence suggests a complex, bidirectional relationship between the two conditions. T2DM is an independent risk factor for accelerated muscle depletion, whereas loss of skeletal muscle, the primary site for postprandial glucose disposal, exacerbates insulin resistance [4,5].

Sarcopenia is a multifactorial condition associated with aging and metabolic disorders involving various pathological mechanisms, including muscle atrophy, impaired myogenesis, and chronic inflammation [6]. Muscle atrophy is a key molecular feature contributing to disease progression. Pro-inflammatory cytokines, including tumor necrosis factor-α (TNF-α) and interleukin-6 (IL-6), along with insulin resistance, promote muscle protein degradation. At the same time, these factors impair the function of muscle satellite cells, thereby inhibiting myogenesis and disrupting muscle homeostasis [7]. Impairment of myogenesis is a critical factor in the progression of sarcopenia.

Recently, there has been increasing interest in therapeutic strategies aimed at promoting muscle regeneration, particularly those based on natural products capable of modulating multiple targets simultaneously [8]. *Momordica charantia* (bitter melon), a member of the *Cucurbitaceae* family, is widely distributed throughout Asia, Africa, and the Caribbean [9]. It has been extensively studied for its antidiabetic and anti-inflammatory properties and has been reported to contribute to the prevention and improvement of metabolic disorders through its glucose-lowering effects, enhancement of insulin signaling, and suppression of oxidative stress and inflammation [10,11].

Although *M. charantia* has not been established as a treatment for sarcopenia, its reported antidiabetic, antioxidant, and anti-inflammatory properties provide a rationale for investigating its potential effects on muscle dysfunction-related cellular responses. Because metabolic dysregulation and inflammation-related signaling are closely associated with impaired myogenic differentiation and muscle atrophy, MCE was evaluated in the present study as a candidate natural product that may modulate dexamethasone-induced sarcopenic changes in C2C12 myotubes.

In this study, network pharmacology was employed to systematically investigate the multicomponent and multitarget characteristics of *M. charantia*. Network pharmacology enables the prediction of potential mechanisms of action underlying complex diseases by analyzing the interactions between bioactive compounds and disease-related targets at the system level. Network pharmacology identified ascorbic acid, tocopherol, and quercetin as major bioactive compounds of *M. charantia*, all of which have been reported to exhibit antioxidant and muscle-protective effects [12,13,14] and were found to be associated with targets related to muscle atrophy and metabolic disorders. Although *M. charantia* has been reported to alleviate muscle atrophy, its direct role in regulating myogenesis remains largely unexplored [15].

Therefore, we hypothesized that MCE, owing to its anti-inflammatory and antidiabetic properties, may selectively suppress pro-inflammatory regulators such as p38 mitogen-activated protein kinase (p38 MAPK) signaling pathway under chronic inflammatory conditions, thereby promoting myotube differentiation without altering protein degradation, ultimately contributing to the amelioration of sarcopenia. The aim of this study was to elucidate the inflammatory regulator-dependent mechanism underlying the pro-myogenic effects of MCE and to propose its potential as a multitarget therapeutic strategy for sarcopenia-associated metabolic disorders through integrated network pharmacology and molecular docking analyses, followed by in vitro validation.

## 2. Results

### 2.1. Gene Ontology (GO) Biological Process Enrichment Analysis of Overlapping Targets of Momordica charantia in Sarcopenia

**Figure 1:**
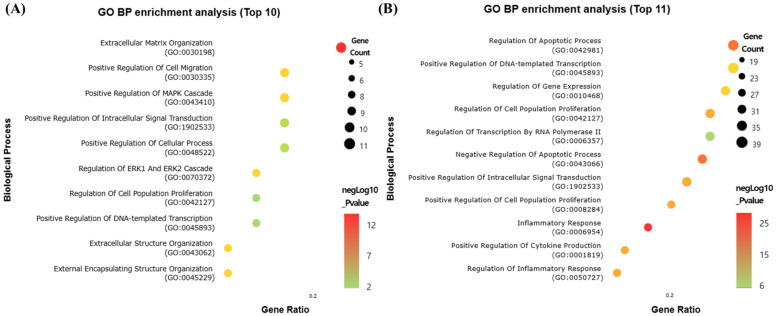
Gene ontology biological process enrichment analysis of overlapping targets of *M. charantia* associated with sarcopenia and metabolic disorders. (**A**) Sarcopenia-related targets. (**B**) Metabolic disorder-related targets. The network illustrates the interactions between bioactive compounds and their predicted targets. Nodes represent compounds and target genes, while edges indicate their interactions. Key hub targets are highlighted based on their degree values.

GO biological process enrichment analysis was performed to explore the biological relevance of overlapping targets of the active compounds present in *M. charantia.* As shown in Figure 1A, sarcopenia-related targets were mainly enriched in extracellular matrix organization, positive regulation of cell migration, positive regulation of the MAPK cascade, positive regulation of intracellular signal transduction, and regulation of cell proliferation. These processes are closely associated with muscle tissue remodeling, intracellular signaling, and regulation of proliferation and migration, suggesting that *M. charantia* may be involved in the regulation of muscle homeostasis and impaired regenerative responses during sarcopenia. Notably, the enrichment of positive regulation of the MAPK cascade supports a possible association with the p38 MAPK-mediated myogenic signaling pathway investigated in the present study.

Since *M. charantia* is widely recognized for its antidiabetic and metabolic regulatory properties, diabetes-related overlapping targets were also analyzed to identify biologically shared processes between sarcopenia and metabolic dysfunction. As shown in Figure 1B, the enriched biological processes included regulation of the apoptotic process, positive regulation of DNA-templated transcription, regulation of gene expression, regulation of transcription by RNA polymerase II, inflammatory response, positive regulation of cytokine production, and regulation of the inflammatory response. These results indicate that the predicted targets of *M. charantia* are involved in biological processes commonly associated with both sarcopenia and metabolic dysfunction, particularly those related to inflammation, transcriptional regulation, and cell survival. Collectively, these findings suggest that *M. charantia* may exert multitarget effects on shared pathological mechanisms linking muscle impairment and metabolic disorders.

### 2.2. Compound–Target–Pathway Network Analysis of M. charantia in Sarcopenia

**Figure 2:**
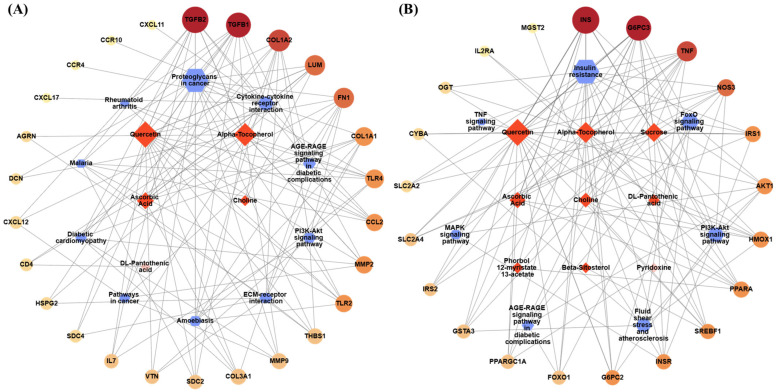
Compound–target–pathway network analysis of *M. charantia* associated with sarcopenia and metabolic disorders. (**A**) Sarcopenia-related network. (**B**) Metabolic disorder-related network. The enrichment analysis shows the significantly associated biological processes and signaling pathways. The *x*-axis represents the gene ratio, and the *y*-axis indicates the enriched terms. The color gradient reflects the adjusted *p*-value.

A compound–target–pathway network was constructed to identify the major active compounds present in *M. charantia* and their predicted targets associated with sarcopenia and metabolic dysfunction (Figure 2). In the sarcopenia-related network (Figure 2A), the major target genes included TGFB1, TGFB2, COL1A2, LUM, FN1, COL1A1, TLR4, CCL2, MMP2, TLR2, THBS1, MMP9, and COL3A1, suggesting their potential involvement in extracellular matrix remodeling, inflammatory regulation, and cytokine-related signaling associated with skeletal muscle impairment. Pathway nodes linked to these targets included the cytokine–cytokine receptor interaction, AGE–RAGE signaling pathway in diabetic complications, PI3K–Akt signaling pathway, and ECM–receptor interaction.

In the metabolic disorder-related network (Figure 2B), the major target genes included INS, G6PC3, TNF, NOS3, IRS1, AKT1, HMOX1, PPARA, SREBF1, INSR, G6PC2, and FOXO1, which were mainly associated with insulin resistance, whereas the major signaling pathways were FOXO, PI3K–Akt, MAPK, and AGE–RAGE pathways, which were associated with diabetic complications. Among the genes, TNF was identified as a functionally relevant inflammatory node, whereas AKT1, FOXO1, and INSR were associated with metabolically relevant intracellular signaling.

Notably, representative compounds such as quercetin, α-tocopherol, and ascorbic acid were connected to multiple targets and pathways in both networks, supporting the multicomponent and multitarget characteristics of *M. charantia*. Given that the sarcopenia-related network included targets associated with extracellular remodeling and inflammatory signaling and that the metabolic disorder-related network included MAPK-related and insulin signaling pathways, subsequent experimental analyses focused on MMP2, MMP9, MAFbx, MyoD, myogenin, and p38 MAPK. Collectively, these findings suggest that *M. charantia* may act on interconnected molecular pathways involved in muscle dysfunction and metabolic imbalance, with the p38 MAPK signaling representing a potential mechanistic axis.

### 2.3. Identification of Putative Compounds in MCE by UPLC-Q-TOF-HRMS

**Figure 3:**
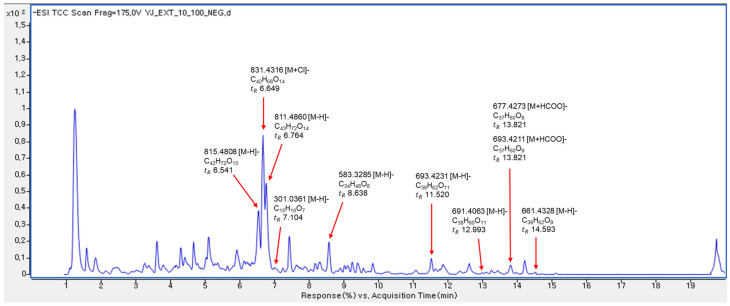
The total compound chromatogram of MCE in negative ion mode by LC-QTOF MS.

Q-TOF-MS is a well-known metabolomics technique that provides high mass accuracy measurements and enables the determination of molecular formulas for compounds. High-resolution MS data offer valuable evidence for the identification of known metabolites by providing molecular information on compounds present in the extracts. This approach is considered particularly powerful because known compounds can be tentatively identified without the use of authentic reference standards. Qualitative analysis of the MCE was performed using UPLC-Q-TOF-HRMS to obtain their chemical profiles, revealing 10 putative compounds, including several cucurbitane-type triterpenoids and quercetin (Table 1 and Figure 3). Based on the available literature, all compounds were compared with the experimental mass data, and the ppm error values were calculated. The compounds were then tentatively characterized according to their mass spectral data. When the difference between the theoretical and measured exact masses was within ±50 ppm, the compound was considered a positive match [16]. The exact masses were determined from the possible deprotonated molecules, chloride adducts, and formate adducts detected in the negative ESI mode.

### 2.4. Effects of M. charantia on Cell Viability and Myotube Morphology

**Figure 4:**
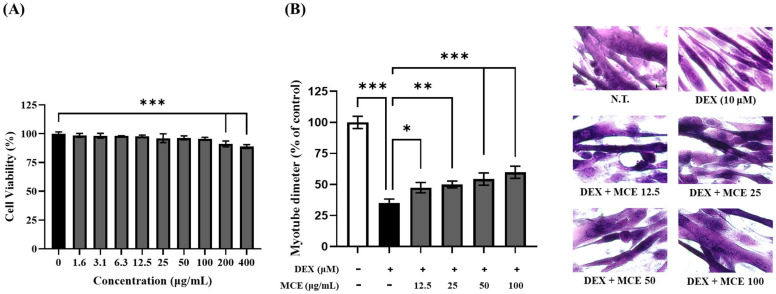
Effects of MCE on cell viability and myotube morphology in DEX-treated C2C12. (**A**) Cell viability following MCE treatment at the indicated concentrations. (**B**) Representative hematoxylin and eosin-stained images and quantitative analysis of myotube morphology in C2C12 treated with DEX with or without MCE. Data are presented as the mean ± standard deviation. MCE—*Momordica charantia* extract; DEX—dexamethasone. * *p* < 0.05, ** *p* < 0.01, and *** *p* < 0.001. The MCE and individual compound experiments were conducted as independent experimental sets, each including its own N.T. and DEX control groups. Therefore, differences in the absolute morphological index values of the DEX-treated groups between experiments may reflect experimental batch-to-batch variation, including differences in C2C12 differentiation efficiency and the degree of DEX-induced myotube impairment. Accordingly, the effects of each treatment were interpreted relative to the corresponding DEX control within the same experimental set. Images were captured at ×400 magnification. Scale bar = 100 μm.

To establish the appropriate concentration range for subsequent experiments, C2C12 cells were treated with MCE, and cell viability was assessed (Figure 4A). Compared with the non-treated (N.T.) group, MCE at concentrations of 1.6–100 μg/mL induced only minimal changes in cell viability, with mean values ranging between 95.6–98.5%. In contrast, cell viability decreased to 91.1% at 200 μg/mL and 88.8% at 400 μg/mL. Accordingly, MCE concentrations up to 100 μg/mL were selected for subsequent experiments. Importantly, the observed variations in cell viability within the 1.6–100 μg/mL range were relatively small and not considered biologically significant, indicating that MCE does not induce significant inhibition of cell viability under the experimental conditions.

To further examine the effects of MCE on myotube morphology, hematoxylin and eosin (H&E) staining was performed under dexamethasone (DEX)-induced conditions (Figure 4B). The DEX-treated group showed a marked reduction in the morphological index to 35.04%, compared with the N.T. group. However, MCE treatment increased the index value in a concentration-dependent manner, reaching 47.5%, 50.0%, 54.4%, and 59.9% at 12, 25, 50, and 100 μg/mL, respectively. Consistent with these quantitative results, representative images showed that DEX impaired myotube morphology, whereas MCE treatment progressively restored myotube formation. These results suggest that the protective effects of MCE are more evident at higher concentrations, where the magnitude of recovery becomes more pronounced.

### 2.5. Effects of M. charantia on Sarcopenia-Related Protein Expression

**Figure 5:**
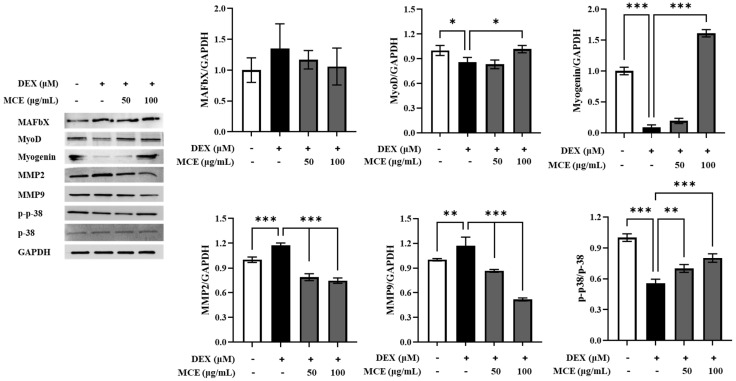
Effects of MCE on sarcopenia-related protein expression in DEX-treated C2C12. Representative Western blot images and quantitative analysis of MAFbX, MMP9, MyoD, myogenin, MMP2, p38, and phosphorylated-p38. Data are presented as the mean ± standard deviation. MCE—*Momordica charantia* extract; DEX—dexamethasone. * *p* < 0.05, ** *p* < 0.01, *** *p* < 0.001.

To investigate the molecular mechanisms underlying the effects of MCE on DEX-induced sarcopenia, protein expression was analyzed by Western blotting. Proteins associated with muscle atrophy, extracellular remodeling, and myogenic differentiation were examined (Figure 5).

The expression of the muscle atrophy-related protein MAFbX increased by 3.8% in the DEX-treated group compared with the N.T. group and further increased by 23.9% in the MCE 50 μg/mL group. In contrast, MAFbX expression decreased by 8.6% in the MCE 100 μg/mL group, indicating attenuation at higher concentration. Similarly, MMP9 expression increased by 17.5% following DEX treatment, whereas MCE treatment reduced its expression by 13.4% at 50 μg/mL and by 48.09% at 100 μg/mL, suggesting that MCE suppressed DEX-induced extracellular remodeling.

Furthermore, expression of myogenic regulatory factors was suppressed by DEX and restored by MCE. MyoD expression decreased by 14.5% in the DEX-treated group compared with the N.T. group. Treatment with MCE at 50 μg/mL showed a 16.8% decrease, whereas treatment with MCE at 100 μg/mL increased MyoD expression by 1.6% relative to the N.T. group. Similarly, myogenin expression decreased by 89.1% following DEX treatment. However, MCE treatment increased myogenin expression, although the 50 μg/mL group still showed an 80.83% decrease relative to the N.T. group, and the 100 μg/mL group demonstrated a 61.0% increase relative to the N.T., indicating marked recovery at the higher concentration.

Moreover, MMP2 expression increased by 17.4% following DEX treatment, whereas MCE treatment reduced its expression by 21.1% at 50 μg/mL and by 25.4% at 100 μg/mL compared with the N.T. group. Notably, phosphorylated p38 expression decreased by 44.3% in the DEX-treated group compared with the N.T. group. MCE treatment partially restored this decrease, with phosphorylated-p38 (p-p38) expression showing a 30.0% decrease at 50 μg/mL and a 19.9% decrease at 100 μg/mL relative to the N.T. group, indicating recovery of p38 MAPK activation.

Overall, while some changes in protein expression were relatively modest in magnitude, more pronounced and biologically meaningful effects were observed at higher concentrations, particularly in the recovery of myogenin expression and the suppression of MMP-2 and MMP-9. These results suggest that MCE exerts dose-dependent regulatory effects on both muscle atrophy-related and myogenic pathways.

Collectively, these results demonstrated that MCE suppressed DEX-induced increases in atrophy- and remodeling-related proteins, including MAFbX, MMP2, and MMP9, while restoring the expression of myogenic factors, including MyoD and myogenin, and enhancing p38 MAPK activation. These findings suggest that MCE alleviates sarcopenia by promoting myogenic differentiation via the p38 MAPK signaling pathway.

### 2.6. Total Flavonoid Content (TFC) of MCE

**Figure 6:**
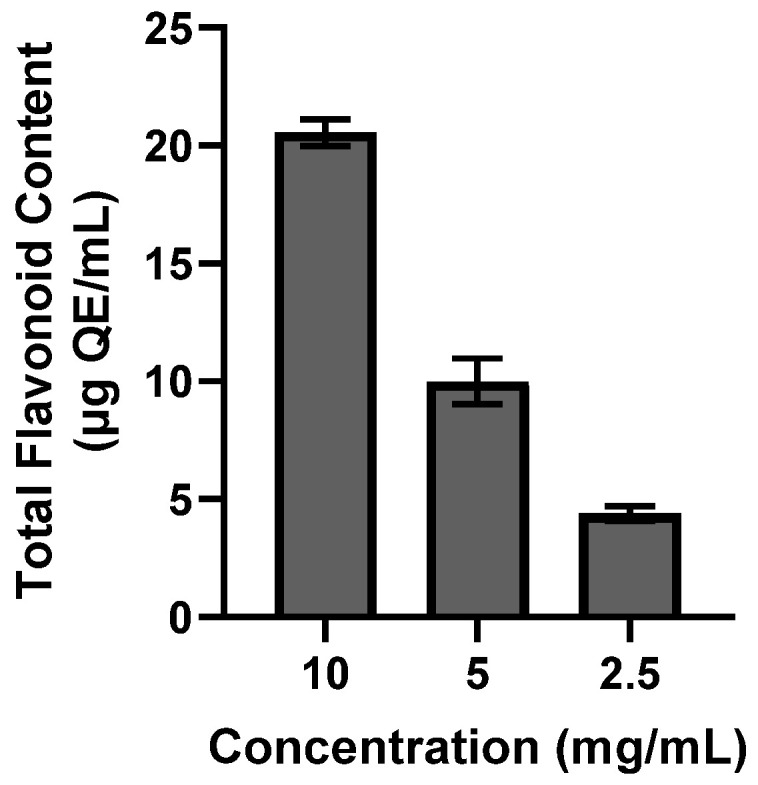
TFC of MCE. TFC was measured at extract concentrations of 10, 5, and 2.5 mg/mL and expressed as μg QE/mL. Values are presented as the mean ± SD (*n* = 3).

The TFC of MCE was measured to evaluate its flavonoid content. As shown in Figure 6, the TFC values of MCE were 20.5, 10.1, and 4.4 μg quercetin equivalents per mL (μg QE/mL) at concentrations of 10, 5, and 2.5 mg/mL, respectively. These results indicate that the measured TFC values increased proportionally with the concentration of MCE.

### 2.7. Effects of Representative Compounds of M. charantia on Cell Viability

**Figure 7:**
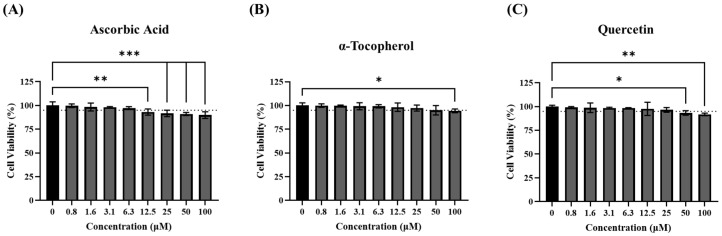
Effects of representative compounds identified from *M. charantia* on cell viability in C2C12. C2C12 cells were treated with (**A**) ascorbic acid, (**B**) α-tocopherol, and (**C**) quercetin at the indicated concentrations, and cell viability was measured. Data are presented as the mean ± SD. * *p* < 0.05, ** *p* < 0.01, *** *p* < 0.001.

To determine the appropriate concentration ranges for subsequent experiments, the effects of representative compounds identified from *M. charantia* on C2C12 cell viability were evaluated (Figure 7). Ascorbic acid, quercetin, and α-tocopherol showed different patterns of cell viability inhibition depending on the treatment concentration.

In the ascorbic acid-treated group (Figure 7A), cell viability was maintained at levels comparable to the N.T. group up to a concentration of 6.3 μM, whereas a reduction in cell viability was observed at concentrations above 12.5 μM. In particular, cell viability decreased to 93.1% at 12.5 μM, corresponding to a 6.9% reduction relative to the N.T. group. Cell viability further decreased to 91.7% at 25 μM, 91.1% at 50 μM, and 89.9% at 100 μM, representing reductions of 8.3%, 8.9%, and 10.09%, respectively. These results indicate a gradual reduction in cell viability; however, the overall magnitude of decrease remains relatively limited within the tested concentration range.

In the α-tocopherol-treated group (Figure 7B), cell viability was largely maintained over a broad concentration range. The mean viability values were 98.3% at 12.5 μM and 97.3% at 25 μM, indicating minimal inhibition at these concentrations. At 50 and 100 μM, cell viability decreased to 95.0% and 94.3%, corresponding to reductions of 5.0% and 5.7%, respectively, relative to the N.T. group. These findings suggest that α-tocopherol does not substantially reduce cell viability under the tested conditions.

In the quercetin-treated group (Figure 7C), cell viability was maintained at levels comparable to those of the N.T. group up to a concentration of 12.5 μM but showed a decreasing trend at concentrations above 25 μM. The mean cell viability was 96.5% at 25 μM, corresponding to a 2.9% reduction relative to the N.T. group. At higher concentrations, cell viability further decreased to 93.3% at 50 μM and 91.8% at 100 μM, representing reductions of 6.1% and 7.6%, respectively. Overall, the observed reductions in cell viability were relatively small and should be interpreted as limited inhibitory effects rather than substantial biological responses.

Taken together, ascorbic acid showed the earliest reduction in cell viability, beginning at 6.3 μM, whereas quercetin and α-tocopherol showed reductions at 50 μM and 100 μM, respectively. Among the tested compounds, α-tocopherol exhibited the weakest effect on cell viability, whereas ascorbic acid showed the greatest reduction at the highest concentration.

### 2.8. Effects of Representative Compounds from M. charantia on Myotube Morphology

To evaluate the effects of the major bioactive compounds of *M. charantia* on myotube morphology under DEX-induced atrophic conditions, C2C12 myotubes were treated with ascorbic acid, α-tocopherol, and quercetin, followed by H&E staining.

Compared with the N.T. group, DEX treatment markedly impaired myotube morphology, as evidenced by a substantial reduction in the morphological index and disrupted myotube structure, confirming the induction of muscle atrophy.

**Figure 8:**
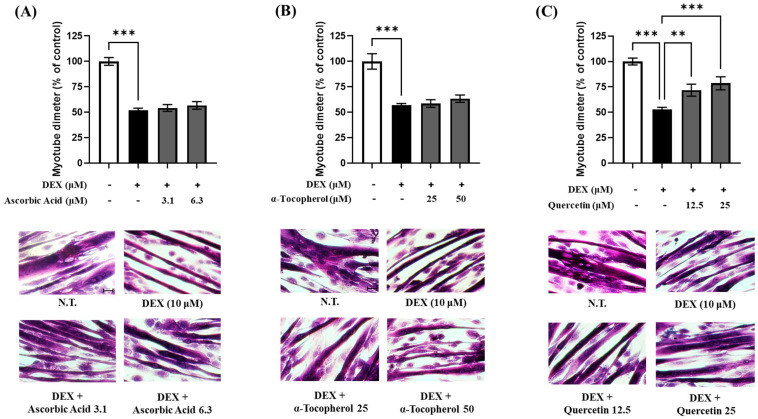
Effects of major compounds present in *M. charantia* on myotube morphology in DEX-treated C2C12. Representative hematoxylin and eosin-stained images and quantitative analysis of myotube morphology following treatment with (**A**) ascorbic acid, (**B**) α-tocopherol, and (**C**) quercetin. Data are presented as the mean ± standard deviation. Images were captured at ×400 magnification. Scale bar = 100 μm. ** *p* < 0.01, and *** *p* < 0.001.

Ascorbic acid treatment slightly reversed the DEX-induced decrease in the morphological index. However, the extent of recovery remained limited, and the morphological index was only increased from 52.1% in the DEX-treated group to 54.2% and 56.7% at ascorbic acid concentrations of 3.1 and 6.3 μM, respectively (Figure 8A). These modest changes suggest only a limited contribution of ascorbic acid to morphological recovery under the tested conditions.

Similarly, α-tocopherol treatment partially attenuated the DEX-induced morphological impairment. The morphological index increased from 57.1% in the DEX-treated group to 58.6% and 63.3% at α-tocopherol concentrations of 12.5 and 25 μM, respectively, indicating a moderate recovery of myotube morphology (Figure 8B). The observed improvements were moderate and more apparent at higher concentrations.

In contrast, quercetin treatment markedly improved DEX-induced myotube atrophy. The morphological index increased from 53.0% in the DEX-treated group to 71.8% and 78.6% at 12.5 and 25 μM of quercetin, respectively, demonstrating a substantially greater recovery compared with ascorbic acid and α-tocopherol (Figure 8C).

These results were consistent with the H&E-stained images and demonstrated that the major compounds present in *M. charantia*, as predicted by network pharmacology analysis, attenuated DEX-induced myotube atrophy. Quercetin showed the most pronounced restorative effect, exhibiting thicker and more elongated myotubes than those treated with DEX.

Collectively, these findings suggest that all major compounds of *M. charantia* contribute to the attenuation of DEX-induced myotube atrophy, with quercetin showing a superior protective effect compared with ascorbic acid and α-tocopherol.

### 2.9. Effects of Representative Compounds from M. charantia on Sarcopenia-Related Protein Expression

To further investigate whether quercetin, one of the bioactive flavonoids present in *M. charantia*, contributes to the protective effects against DEX-induced muscle atrophy, Western blot analysis was performed in C2C12 myotubes.

**Figure 9:**
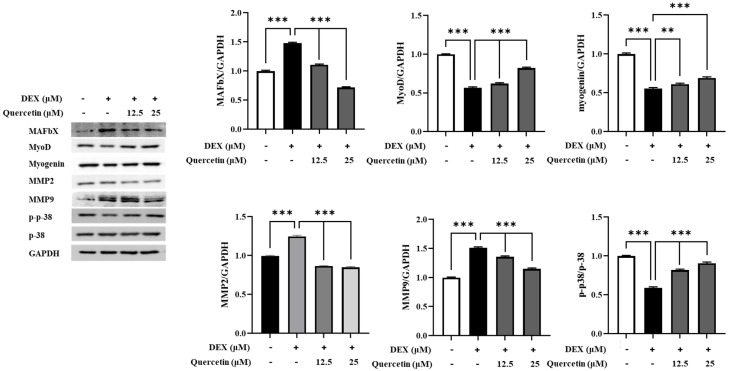
Effects of quercetin from *M. charantia* on sarcopenia-related protein expression in DEX-treated C2C12. Representative Western blot images and quantitative analysis of MAFbX, MMP9, MyoD, myogenin, MMP2, p38, and phosphorylated-p38. Data are presented as the mean ± standard deviation. MCE—*Momordica charantia* extract; DEX—dexamethasone. ** *p* < 0.01, *** *p* < 0.001.

DEX treatment markedly increased the expression of muscle atrophy-related proteins. The expression level of MAFbx was increased by 48.2% in the DEX-treated group compared with the non-treated control group. However, quercetin treatment reduced the DEX-induced increase in MAFbx expression by 24.9% and 51.5% at 12.5 and 25 μM, respectively, compared with the DEX-treated group. Similarly, MMP9 expression was increased by 51.3% following DEX treatment, whereas quercetin decreased MMP9 expression by 10.2% and 23.9% at 12.5 and 25 μM, respectively. MMP2 expression was also increased by 25.1% in the DEX-treated group compared with the control group, and this increase was attenuated by quercetin treatment, with reductions of 30.6% and 31.8% at 12.5 and 25 μM, respectively (Figure 9).

In contrast, DEX treatment reduced the expression of myogenic differentiation-related proteins. MyoD expression was decreased by 43.2% in the DEX-treated group compared with the non-treated control group. Quercetin treatment partially restored MyoD expression, increasing it by 9.5% and 44.8% at 12.5 and 25 μM, respectively, compared with the DEX-treated group. Myogenin expression was also reduced by 44.3% following DEX treatment, whereas quercetin increased myogenin expression by 9.8% and 24.3%, compared with the DEX-treated group.

Furthermore, the p-p38/p38 ratio was decreased by 41.0% in the DEX-treated group compared with the non-treated control group. Quercetin treatment restored the DEX-suppressed p-p38/p38 ratio by 38.5% and 53.9% compared with the DEX-treated group.

Collectively, these results indicate that quercetin attenuated DEX-induced muscle atrophy-related changes by suppressing the upregulation of MAFbx, MMP9, and MMP2, while restoring the expression of myogenic markers, including MyoD and myogenin. These findings suggest that quercetin may contribute to the protective effect of *M. charantia* against DEX-induced muscle atrophy in C2C12 myotubes.

### 2.10. Binding Affinity, Pose Convergence, and Mode Selection

Molecular docking of the selected ligands against the p38α (MAPK14) target revealed distinct binding energy profiles. Based on the RMSD–energy correlation criteria across the 50-mode trajectories, α-tocopherol was identified as the most potent hit, yielding a highly exergonic binding energy of −8.685 kcal/mol for its primary conformation (Mode 1), coupled with ideal RMSD. Quercetin demonstrated a comparable high-affinity binding profile with a primary Mode 1 and exhibited a docking score of −8.414 kcal/mol, without a RMSD variance. Conversely, ascorbic acid exhibited significantly attenuated target affinity, characterized by a binding energy of −5.327 kcal/mol (Mode 1, RMSD = 0.000 Å). Vina pose convergence topology confirmed that the primary docking modes of both α-tocopherol and quercetin were energetically segregated from their alternative 49 conformations and tightly clustered around Mode 1, indicating highly stable and reproducible binding conformations for these ligands.

### 2.11. Interaction Profiles of Compounds

**Figure 10:**
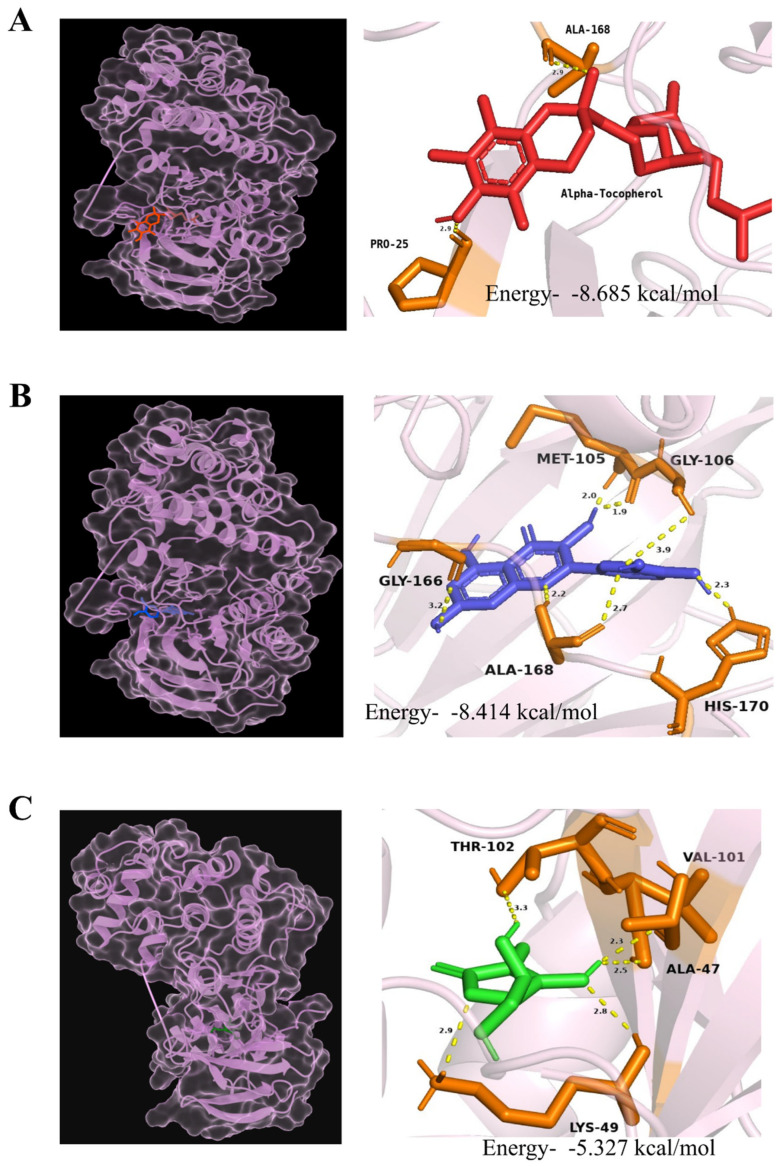
Binding of selected compounds at the p38α active site. The binding of shortlisted compounds, α-tocopherol, quercetin, and ascorbic acid, within the p38α active site is shown in panels (**A**), (**B**), and (**C**), respectively. The left panel illustrates the macromolecular view, and the right panel depicts key polar interactions with the p38α residues at the binding site, represented by yellow dashed lines. The interatomic distances between interacting atoms are indicated in angstrom (Å), and the corresponding binding energy of each compound is provided in kcal/mol.

Protein–ligand interaction profiler (PLIP) architectural analysis revealed that the optimal binding affinity of α-tocopherol (−8.685 kcal/mol) is primarily driven by an extensive network of hydrophobic interactions that effectively anchor its lipophilic tail within the reference ligand’s cavity. The ligand engages in critical hydrophobic interactions with 10 distinct amino acid residues, as listed in Table 2. This dominant hydrophobic envelope is conformationally rigidified by two targeted hydrogen bonds involving the chromanol ring oxygen moieties (Figure 10A).

While α-tocopherol relies on hydrophobic desolvation, it achieves its high-affinity binding state (−8.414 kcal/mol) through a highly directional array of polar interactions. The polyhydroxylated flavonoid scaffold forms a robust hydrogen-bonding network with MET-105, GLY-106, GLY-166, ALA-168, and HIS-170 (Figure 10B). The short interatomic distances observed with MET-105 (2 Å) and ALA-168 (2.2 Å) indicate strong, highly stabilizing dipole–dipole interactions critical for maintaining ligand occupancy within the binding cavity.

In contrast, a reduced binding affinity was observed for ascorbic acid (−5.327 kcal/mol), which correlates directly with its spatially restricted interaction footprint. Its binding is primarily mediated through hydrogen bonding with residues ALA-47, LYS-49, VAL-101, and THR-102 (Figure 10C). The distinct lack of a complementary hydrophobic interaction network likely restricts its conformational stability within the p38α pocket, resulting in the observed thermodynamic penalty of 3.358 kcal/mol relative to α-tocopherol.

## 3. Discussion

In this study, the effects of MCE on sarcopenia-related changes were investigated using an integrated approach combining network pharmacology, C2C12 cell-based experiments, Western blotting, and molecular docking analysis. Network pharmacology analysis identified the p38 MAPK signaling pathway and potential bioactive candidates, including quercetin, ascorbic acid, and tocopherol, as being associated with muscle atrophy and metabolic disorders (Figure 1 and Figure 2). These findings suggest that *M. charantia* may influence multiple molecular processes involved in sarcopenia-related cellular dysfunction.

*M. charantia* has traditionally been used to treat diabetes and metabolic disorders and has been reported to exhibit diverse pharmacological activities, including antioxidant and anti-inflammatory effects [17,18,19]. These biological properties are closely associated with key pathological mechanisms of sarcopenia, such as oxidative stress and chronic inflammation, thereby supporting its potential relevance in muscle-related disorders. Notably, sarcopenia is characterized not only by increased protein degradation but also by impaired myogenesis; thus, promotion of muscle differentiation is considered an important strategy for maintaining skeletal muscle homeostasis [15,20,21].

While previous studies have mainly focused on the antioxidant, anti-inflammatory, and antidiabetic properties of *M. charantia*, the present study extends these findings by linking MCE to myogenic differentiation and extracellular matrix remodeling in DEX-induced sarcopenic conditions. In particular, the combined changes in p38 MAPK-related signaling, MyoD, myogenin, MAFbx, MMP-2, and MMP-9 suggest that MCE may influence both regenerative and remodeling-associated responses rather than acting solely through general antioxidant or anti-inflammatory mechanisms. Therefore, the scientific novelty of this study lies in proposing the p38 MAPK-associated myogenic differentiation axis and ECM remodeling as potential mechanistic features underlying the effects of MCE in sarcopenia-related cellular changes.

Myogenesis involves the activation and differentiation of satellite cells into myotubes. The p38 MAPK signaling pathway plays a crucial role in regulating the transcriptional activity of myogenic factors, including MyoD and myogenin [22]. Under chronic inflammatory and diabetic conditions, this pathway can be dysregulated, leading to impaired muscle formation [23,24,25,26]. Additionally, MMP-2 and MMP-9 contribute to extracellular matrix remodeling and inflammation, thereby impairing satellite cell function and ultimately promoting muscle atrophy [27,28]. Therefore, modulation of myogenic signaling and extracellular matrix remodeling may be relevant for improving sarcopenia-related muscle impairment.

Skeletal muscle homeostasis is maintained through a balance between protein synthesis and degradation. Current therapeutic strategies primarily focus on inhibiting protein degradation via the ubiquitin–proteasome system, particularly through regulation of MAFbx/Atrogin-1 [29,30,31]. Although these approaches may reduce muscle loss, they have limitations in restoring impaired muscle regeneration. In this context, the present study focused on whether MCE could support myogenic differentiation under DEX-induced atrophic conditions.

In C2C12 cells, MCE did not meaningfully reduce cell viability within the concentration range selected for subsequent experiments, while improving DEX-induced impairment of myotube morphology. These findings suggest that MCE may support myotube formation under atrophic conditions without markedly inhibiting cell viability. The changes in p-p38, MyoD, myogenin, MAFbx, MMP-2, and MMP-9 collectively suggest that MCE may influence both myogenic differentiation and remodeling-associated responses in DEX-induced sarcopenic conditions (Figure 5). In particular, the recovery of myogenic regulatory factors together with the suppression of MMP-2 and MMP-9 supports the possibility that MCE contributes to the preservation of a cellular environment favorable for myotube formation.

Although the observed restoration of phosphorylated p38 and myogenic regulatory factors suggests the involvement of the p38 MAPK signaling pathway, the present study does not provide direct functional evidence to establish a causal relationship. In particular, the absence of pathway-specific inhibition or gene silencing approaches limits the ability to confirm whether the observed effects are directly mediated through p38 MAPK activation. Therefore, the current findings should be interpreted as indicative of pathway involvement rather than definitive proof of causality, and further studies employing specific inhibitors or genetic modulation strategies will be necessary to clarify the precise mechanistic role of this pathway.

Among the identified compounds, quercetin, ascorbic acid, and tocopherol are known to exert antioxidant and anti-inflammatory effects that support muscle cell protection and differentiation [32]. These compounds may contribute to the regulation of muscle-related pathways by alleviating oxidative stress and inflammation [25,33,34]. Morphological analysis revealed that while ascorbic acid and α-tocopherol showed non-significant recovery trends, quercetin significantly improved myotube morphology in a dose-dependent manner (Figure 7 and Figure 8). These findings suggest that quercetin may be one of the representative contributors to the observed effects, while the overall activity of MCE is likely influenced by multicomponent interactions.

In addition to the qualitative identification of quercetin by UPLC-Q-TOF-HRMS, the total flavonoid content of MCE was measured to further characterize its flavonoid-associated chemical properties. The TFC values increased proportionally with the concentration of MCE, indicating that flavonoid constituents are present in the extract in a concentration-dependent manner. This result provides additional chemical support for the biological relevance of flavonoid-related compounds in MCE. However, because TFC represents the overall flavonoid-equivalent content rather than the amount of a single compound, these findings should be interpreted as evidence of flavonoid enrichment rather than direct quantification of quercetin itself.

Consistent with the morphological recovery observed in quercetin-treated myotubes, Western blot analysis further supported the contribution of quercetin to the protective effects of MCE against DEX-induced sarcopenic changes. Quercetin suppressed the DEX-induced upregulation of MAFbx, MMP-9, and MMP-2, suggesting attenuation of muscle atrophy-related and extracellular remodeling responses. In parallel, quercetin restored the expression of MyoD and myogenin, indicating recovery of myogenic differentiation-related signaling. Moreover, the DEX-induced decrease in the p-p38/p38 ratio was reversed by quercetin treatment, supporting the involvement of p38 MAPK-associated myogenic signaling. These findings suggest that quercetin may be one of the representative flavonoid constituents contributing to the anti-sarcopenic effects of MCE, particularly through the coordinated regulation of atrophy-related proteins, extracellular remodeling markers, and myogenic regulatory factors.

Quercetin is widely distributed in various medicinal and edible plants; therefore, its presence alone cannot fully explain the specific biological effects of MCE. The protective effect observed in the present study is more likely to result from the combined actions of multiple constituents rather than the activity of a single compound. In complex botanical extracts, bioactive compounds may interact synergistically or additively by modulating overlapping pathways related to oxidative stress, inflammation, extracellular matrix remodeling, and myogenic differentiation. Thus, although quercetin showed a relatively pronounced effect in the individual compound experiments, it should be regarded as one of the representative candidate compounds contributing to the overall activity of MCE, rather than as the sole determinant of its anti-sarcopenic effect.

Although network pharmacology analysis identified quercetin, ascorbic acid, and tocopherol as potential bioactive compounds associated with anti-sarcopenic targets, only a subset of these compounds demonstrated pronounced efficacy in the in vitro model. This discrepancy may be attributed to several factors. Firstly, network pharmacology predictions are primarily based on database-derived compound–target interactions and do not fully account for pharmacokinetic properties such as cellular uptake, stability, and intracellular bioavailability. For instance, ascorbic acid and tocopherol may exhibit limited stability or reduced effective intracellular concentrations under experimental conditions [35,36], thereby attenuating their observable biological activity in C2C12 cells. Secondly, the predicted targets may require synergistic interactions among multiple compounds present in the whole extract, which cannot be recapitulated when individual compounds are tested in isolation. This is particularly relevant for complex botanical extracts, where multicomponent and multitarget interactions contribute to overall efficacy [37,38]. Third, differences in experimental context, including the use of a dexamethasone-induced sarcopenia model, may influence pathway activation in a manner that selectively favors certain compounds, such as quercetin, over others. Finally, the network pharmacology approach does not consider compound-specific differences in target binding kinetics or downstream signaling modulation within the cellular environment, which may further contribute to the observed divergence [39,40].

Although the compound–target–pathway network was initially constructed based on database-derived phytochemical information, the chemical composition of the MCE used in this study was further characterized by UPLC-Q-TOF-HRMS. The analysis revealed several putative compounds, including cucurbitane-type triterpenoids and quercetin, which are consistent with previously reported phytochemical constituents of *M. charantia.* In addition, TFC analysis provided further support for the presence of flavonoid-associated constituents in MCE. Nevertheless, the identified compounds should still be interpreted as putative constituents because the analysis was based on high-resolution mass spectral data without confirmation using authentic standards. Moreover, phytochemical profiles of plant extracts may vary depending on geographical origin, maturity, physiological stage, and extraction conditions. Therefore, further targeted LC-MS/MS-based quantification and comparative analysis with previously reported *M. charantia* extracts will be necessary to define the complete metabolite composition of MCE and to clarify how individual compounds and their combinations contribute to its biological activity [41,42,43,44].

While network pharmacology provides a valuable framework for identifying potential multitarget interactions, factors such as compound bioavailability, cellular uptake, metabolic stability, target accessibility, and pathway crosstalk may contribute to the differences between in silico predictions and in vitro results. These observations suggest that the discrepancy between network pharmacology predictions and in vitro outcomes is not necessarily contradictory but rather reflects the inherent differences between theoretical target-based predictions and experimentally observed biological responses. In silico prediction may not always accurately reflect the complex biological responses occurring under actual experimental conditions. Therefore, the combined interpretation of in silico and experimental results should be considered complementary rather than conflicting, providing a more comprehensive understanding of the biological effects of MCE.

To further elucidate the potential molecular interactions underlying the observed effects, molecular docking analysis targeting p38α was performed. The results showed that α-tocopherol and quercetin exhibited relatively strong binding affinity toward p38α, suggesting their potential association with p38 MAPK-related signaling (Figure 10A). α-Tocopherol predominantly interacted through hydrophobic contacts, whereas quercetin formed a hydrogen-bonding network, indicating distinct binding characteristics (Figure 10B). In contrast, ascorbic acid displayed a relatively weak binding affinity, likely due to its limited hydrophobic interactions within the binding pocket (Figure 10C). Given the critical role of p38 MAPK in regulating myogenic differentiation, these results suggest that quercetin and α-tocopherol may be associated with p38-related signaling, whereas ascorbic acid may exert its effects mainly through indirect pathways such as antioxidative effects.

Collectively, these findings suggest that MCE may exert multitarget effects through interactions among multiple bioactive candidates, consistent with the multicomponent and multitarget paradigms of network pharmacology. This approach may be relevant for complex conditions such as sarcopenia, in which impaired myogenesis, extracellular matrix remodeling, oxidative stress, and inflammation are interconnected. To further support the inflammation-related relevance of MCE, NO production was additionally evaluated in LPS-stimulated RAW 264.7 macrophages and included in the Supplementary Figure S1. This supplementary assay provides supportive evidence that MCE and quercetin may modulate inflammatory mediator production in an inflammatory cell model. However, both the RAW 264.7 NO production assay and the DEX-induced C2C12 myotube atrophy model remain simplified in vitro systems and do not fully reproduce the complex and chronic inflammatory environment associated with sarcopenia in vivo. Therefore, the present findings should be interpreted as preliminary cell-based evidence rather than direct proof of therapeutic efficacy in chronic inflammatory sarcopenia. Future studies using appropriate in vivo muscle wasting models are required to validate the physiological relevance, bioavailability, safety, and therapeutic potential of MCE.

Although comparison with a reference drug would strengthen the pharmacological interpretation of this study, there is currently no approved or universally accepted reference drug specifically established for sarcopenia or for the DEX-induced C2C12 myotube atrophy model. Therefore, future studies should include clinically relevant comparators or standardized reference agents, when available, to further evaluate the relative efficacy and therapeutic potential of MCE.

Unlike approaches that primarily focus on suppressing protein degradation, the present findings suggest that supporting myogenic differentiation may be a relevant strategy for improving sarcopenia-related cellular changes. However, further mechanistic, phytochemical, and in vivo studies are required before this approach can be translated into a therapeutic application.

## 4. Materials and Methods

### 4.1. Network Pharmacology Analysis

#### 4.1.1. Scope of Analysis

In the present study, a network pharmacology approach was employed to investigate the potential mechanisms underlying the anti-sarcopenic effects of *M. charantia*. Active compounds were identified, their putative targets were predicted, and sarcopenia-related genes were identified. Overlapping targets were used to construct an interaction network and perform GO biological process enrichment analysis, thereby exploring the multicomponent and multitarget characteristics of *M. charantia* in the context of sarcopenia.

#### 4.1.2. Collection and Preprocessing of Bioactive Compounds

The bioactive compounds of *M. charantia* were collected from the TM-MC and COCONUT databases. The structural information of each compound was organized in SMILES format, and chemical structures were verified using the PubChem database. Putative target proteins for each compound were collected from the STITCH, TM-MC, and ChEMBL databases. Redundant entries were removed, gene names were standardized, and an integrated compound–target dataset was constructed for subsequent analysis.

#### 4.1.3. Identification of Disease-Associated Targets

Sarcopenia-related genes were obtained from the Comparative Toxicogenomics Database. Genes associated with muscle atrophy, impaired myogenesis, chronic inflammation, and insulin resistance were preferentially selected to reflect the key pathological characteristics of sarcopenia. Overlapping genes between the predicted targets of *M. charantia* compounds and sarcopenia-related genes were identified as potential therapeutic targets.

#### 4.1.4. Network Construction and Topological Analysis

Overlapping targets were used to construct an interaction network linking bioactive compounds, target genes, and sarcopenia-related pathways. The network was visualized using Cytoscape.js (v3.34.0) to examine the potential relationships among compounds, targets, and biological functions associated with sarcopenia.

#### 4.1.5. GO and KEGG Pathway Enrichment Analysis

GO biological process enrichment analysis was performed on the overlapping target genes using the Enrichr platform (https://maayanlab.cloud/Enrichr/, accessed on 1 March 2026). Statistical significance was set at *p* < 0.05. 3. The enriched biological processes were interpreted primarily in the context of muscle cell differentiation, muscle development, inflammatory responses, oxidative stress, metabolic regulation, and insulin signaling.

### 4.2. UPLC-Q-TOF MS/MS Analysis for MCE

UPLC–Q–TOF MS/MS analysis was performed using an Agilent G6545B quadrupole time-of-flight (Q–TOF) mass spectrometer (Agilent Technologies, Santa Clara, CA, USA) coupled to an Agilent 1260 Infinity II system. The *M. charantia* extract was weighed and dissolved in 100% methanol, and then diluted with a methanol-water (1:1, *v*/*v*) mixture and filtered through a 0.45 µm hydrophobic polytetrafluoroethylene (PTFE) membrane filter prior to analysis. A 10 µL aliquot of the *M. charantia* extract (4000 ppm) was injected onto an ACQUITY UPLC BEH C18 Column (2.1 mm × 150 mm; 1.7 μm; Waters Corp., Milford, MA, USA) maintained at 30 °C. The mobile phase consisted of 0.1% (*v*/*v*) formic acid in water (A) and 0.1% (*v*/*v*) formic acid in methanol (B), delivered at a flow rate of 0.3 mL/min under the following gradient conditions: 10–100% B over 12 min, maintained isocratically at 100% B for 4 min, and re-equilibrated at 10% B for 4 min. The MS and MS/MS parameters were set as follows: negative ionization mode; gas temperature, 320 °C; drying gas (N_2_) flow rate, 8 L/min; nebulizer pressure, 35 psi; sheath gas temperature, 350 °C; sheath gas flow rate, 11 L/min; capillary voltage, 3500 V; nozzle voltage, 1000 V; fragmentor voltage, 150 V; MS range, *m*/*z* 100–1700; MS acquisition rate, 1 spectrum/s; acquisition time, 1000 ms/spectrum; MS/MS range, *m*/*z* 50–500; MS/MS acquisition rate, 1 spectrum/s; acquisition time, 1000 ms/spectrum; and fixed collision energy, 40 eV. Internal reference compounds (purine and HP-0921) were used for real-time mass calibration. The reference ions in the negative ion mode were observed at *m*/*z* 112.9855 and 1033.9881.

### 4.3. Plant Materials

MCE was prepared from the fruit of *M. charantia*. Fully matured fruits were harvested, post-ripened for 15 days, and then dried. The seeds were removed to obtain the fruit pulp prior to extraction. The powder was then mixed with 50% aqueous ethanol at a ratio of 1:1 (*w*/*v*), followed by ultrasonic extraction using an ultrasonic cleaner (UCP-10; Jeio Tech Co., Ltd., Daejeon, Korea) at 60 °C for 30 min. The extraction process consisted of 30 min of sonication followed by a 10 min rest period and was repeated three times. After extraction, the mixture was centrifuged at 5000 rpm for 10 min, and the supernatant was collected. The obtained extract was subsequently freeze-dried and stored until further use. The freeze-dried MCE was dissolved in dimethyl sulfoxide prior to use.

### 4.4. Cell Line and Materials

The mouse-derived myoblast cell line C2C12 was obtained from the American Type Culture Collection (Manassas, VA, USA). For cell culture and differentiation, Dulbecco’s modified Eagle’s medium (DMEM) was purchased from Sigma-Aldrich (St. Louis, MO, USA), horse serum (HS) was purchased from Gibco (Gaithersburg, MD, USA), and fetal bovine serum (FBS) and penicillin–streptomycin (P/S) were purchased from Thermo Fisher Scientific (Waltham, MA, USA). Phosphate-buffered saline (PBS) was purchased from WELGENE (GyeongSan, Korea). DEX, H&E, and formaldehyde solution were purchased from Sigma-Aldrich (St. Louis, MO, USA). For protein expression analysis, the primary antibodies p-p38 MAPK, p38 MAPK, MyoD, and myogenin, the secondary anti-rabbit antibody conjugated with glyceraldehyde 3-phosphate dehydrogenase (GAPDH), and horseradish peroxidase (HRP) were purchased from Cell Signaling Technology (Danvers, MA, USA). Ascorbic acid, α-tocopherol, and quercetin were used as representative standard compounds of *M. charantia* and were purchased from Sigma-Aldrich (St. Louis, MO, USA).

### 4.5. Cell Culture and Myogenic Differentiation

C2C12 mouse myoblasts were cultured in DMEM supplemented with 10% FBS and 1% P/S (100 U/mL) under humidified conditions at 37 °C in an atmosphere of 5% CO_2_. To induce myogenic differentiation, the culture medium was replaced with DMEM containing 2% HS and 1% P/S, and the cells were incubated for 7 days. During the differentiation period, DEX (10 μM) and MCE (50 and 100 μg/mL) were added to the medium either alone or in combination.

### 4.6. Cell Viability Assay

C2C12 cells were seeded in 96-well plates at a density of 5.0 × 10^4^ cells/mL and treated with varying concentrations of MCE (0–400 μg/mL) for 24 h. Cell viability was subsequently determined using an EZ-Cytox cell viability assay kit (DoGenBio, Seoul, Korea) [45] according to the manufacturer’s instructions.

### 4.7. H&E Staining

To evaluate morphological changes in myotubes, H&E staining [46] was performed on day 7 of differentiation. Cells were washed with PBS and fixed in 4% formaldehyde solution for 30 min, followed by washing with PBS. The cells were then stained with hematoxylin solution for 3 min and rinsed under running distilled water. Subsequently, counterstaining was performed using eosin solution for 1 min. H&E-stained myotubes were observed under an optical microscope, and myotube length was quantified using ImageJ software (ver. 1.53; National Institutes of Health, Bethesda, MD, USA). Morphological quantification was performed independently for each experimental set, and treatment effects were evaluated relative to the corresponding DEX-treated control group within the same experiment.

### 4.8. Western Blotting

On day 7 of differentiation, C2C12 cells were washed with PBS and lysed using radioimmunoprecipitation assay buffer (50 mM Tris–HCl, pH 7.4; 150 mM NaCl; 0.25% sodium deoxycholate; 1% NP-40; and 1 mM EDTA). Protein concentration was determined using a BCA protein assay kit according to the manufacturer’s instructions. Equal amounts of protein were separated by sodium dodecyl sulfate–polyacrylamide gel electrophoresis and transferred onto polyvinylidene difluoride membranes (Merck Millipore, Darmstadt, Germany) [47]. The membranes were blocked with 5% skim milk in Tris-buffered saline containing Tween-20 (TBST; 20 mM Tris, pH 7.4; 150 mM NaCl; and 0.1% Tween-20) for 1 h at room temperature. The membranes were then incubated with primary antibodies overnight at 4 °C, followed by washing with TBST and incubation with HRP-conjugated secondary antibodies for 1 h at room temperature. Protein bands were visualized using an ECL Plus Western blot detection reagent (GE Healthcare, Piscataway, NJ, USA) and detected using a FUSION Solo chemiluminescence imaging system (PEQLAB Biotechnologie GmbH, Erlangen, Germany). Band intensities were quantified using ImageJ software and normalized to GAPDH.

### 4.9. Determination of TFC

TFC was determined based on the formation of a complex between aluminum chloride (Sigma-Aldrich; St. Louis, MO, USA) and flavonoids. Briefly, 0.1 mL of MCE was mixed with 0.56 mL of distilled water and 0.3 mL of 99.5% (*v*/*v*) ethanol. Subsequently, 0.02 mL of 1 M potassium acetate and 0.02 mL of 10% (*w*/*v*) aluminum chloride were added to the mixture, followed by incubation at room temperature for 30 min. The absorbance was measured at 415 nm using a spectrophotometer. A calibration curve was constructed using quercetin as the standard, and the TFC was expressed as μg quercetin equivalents per mL (μg QE/mL).

### 4.10. Molecular Docking Studies

#### 4.10.1. Ligand Cheminformatics and Preparation Pipeline

A structural library of shortlisted compounds was initially curated as SMILES strings obtained from the PubChem and subsequently converted into 3D SDF formats using a custom automated Python (Version: 3.13.12) pipeline based on the RDKit 2026.03.2 cheminformatics suite. Initial 3D molecular coordinates were generated using the ETKDGv3 empirical dispersion algorithm for conformer generation. This process was followed by rigorous geometric optimization and energy minimization using the Universal Force Field. To ensure structural viability and preclude docking failures, a stringent steric clash filter was implemented, systematically rejecting conformers exhibiting interatomic distances below a threshold of 0.85 Å. The validated SDF structures were subsequently protonated at physiological pH (7.4) using OpenBabel v3.1.1. The final structures were parameterized and refined with partial charge assignment and converted into the requisite PDBQT format using the *prepare_ligand4.py script* available in MGLTools version 1.5.7.

#### 4.10.2. Receptor Preparation and Grid Delineation

The macromolecular structure of the target kinase, p38α (MAPK14, PDB ID: 5WJJ), was meticulously prepared using the Dock Prep module within UCSF ChimeraX v1.11.1 [48]. This procedure involves algorithmic addition of missing hydrogen atoms, assignment of appropriate protonation states, calculation of partial charges, and structural remediation of truncated side chains. The active site was explicitly delineated based on the spatial occupancy of the co-crystallized reference ligand. A rigid Cartesian grid box was subsequently generated and mapped using the DockingPie v1.2 plugin within PyMOL [49]. The grid was constructed to encompass the targeted binding pocket, defined with isotropic dimensions of 24.0 × 24.0 × 24.0 Å and a spatial center coordinate of X = 15.85, Y = −7.29, and Z = 4.91.

#### 4.10.3. Molecular Docking Protocol and Pose Selection

Molecular docking simulations were performed using the AutoDock Vina v1.2.7 program [50]. To maximize conformational space sampling within the defined topological grid, the search exhaustiveness parameter was increased to 32. The heuristic search algorithm generated a conformational ensemble of 50 binding modes per ligand, which were filtered using an energy range threshold of 3.0 kcal/mol.

To rigorously determine the definitive binding conformation, systematic bivariate correlation analysis was applied to the 50-mode ensemble. The definitive binding mode was established by correlating the most exergonic binding free energy (kcal/mol) with the absolute minimum RMSD, considering both lower and upper bounds. The final conformation was selected based on the minimum RMSD variance relative to the energetically optimal poses.

#### 4.10.4. Interaction Profiling and Structural Visualization

Post docking pose interaction profiling was performed using the PLIP web server (plip-tool.biotec.tu-dresden.de) to systematically extract and characterize non-covalent topologies, including hydrogen bonding networks, hydrophobic contacts, and dipole interactions. Comprehensive three-dimensional macromolecular visualization and high-resolution binding site architecture analyses were performed using UCSF ChimeraX and PyMOL (TM) Molecular Graphics System (ver. 3.1.0; Schrödinger, LLC).

### 4.11. Statistical Analysis

All experiments were independently performed at least three times. Data are presented as mean ± standard error of the mean. Statistical analyses were performed using the GraphPad Prism (ver. 10.6.1; GraphPad Software Inc., San Diego, CA, USA). Differences among groups were analyzed using one-way analysis of variance, followed by Tukey’s post hoc test. Statistical significance was set at *p* < 0.05.

## 5. Conclusions

MCE attenuated DEX-induced sarcopenic changes in C2C12 cells by improving myotube morphology and modulating the expression of proteins associated with muscle atrophy, extracellular matrix remodeling, and myogenic differentiation. In particular, the extract reduced MAFbx, MMP-2, and MMP-9 expression while restoring p38 MAPK activation and upregulating MyoD and myogenin expression, suggesting that its protective effects may be associated with the recovery of myogenic signaling. Network pharmacology analysis identified quercetin, ascorbic acid, and tocopherol as potential bioactive compounds associated with targets relevant to sarcopenia and metabolic dysfunction. Subsequent compound-based experiments showed that these compounds partially contributed to the attenuation of DEX-induced myotube atrophy, with quercetin showing the most pronounced restorative effect. Molecular docking further suggested potential interactions of these compounds with p38α. Taken together, these findings indicate that MCE may modulate sarcopenia-related changes through multicomponent and multitarget mechanisms, particularly by influencing myogenic differentiation and p38 MAPK-associated signaling. Further studies, including phytochemical profiling, functional pathway validation, and in vivo experiments, are required to confirm the biological relevance and translational potential of MCE.

## Figures and Tables

**Table 1:** Chemical profile of MCE by UPLC-QTOF-HRMS.

Tentative Compounds	Retention Time (min)	Formula	Theoretical (*m*/*z*)	Experimental (*m*/*z*)	Deprotonated Ones or Adducts	Δ (ppm) ^a^
(3β,9β,10α,22*S*,23*R*,24*R*)-22,23,24-Trihydroxy-9-methyl-19-norlanost-5-ene-3,25-diyl bis[β-D-galactopyranoside]/Momorcharaside A	6.541	C_42_H_72_O_15_	815.4798	815.4808	[M-H]^−^	1.23
Momordicine II 7-*O*-glucopyranoside	6.649	C_42_H_68_O_14_	831.4303	831.4316	[M+Cl]^−^	1.56
Karaviloside V	6.764	C_43_H_72_O_14_	811.4844	811.4860	[M-H]^−^	1.97
Quercetin	7.104	C_15_H_10_O_7_	301.0354	301.0361	[M-H]^−^	2.33
(9β,10α,16α,23*E*)-25-(Acetyloxy)-2-ethoxy-16,20-dihydroxy-9-methyl-19-norlanosta-1,5,23-triene-3,11,22-trione	8.638	C_34_H_48_O_8_	583.3276	583.3285	[M-H]^−^	1.54
Scrophoside A	11.520	C_38_H_62_O_11_	693.4219	693.4231	[M-H]^−^	1.73
(2β,3β,9β,10α,16α,23*E*)-25-(Acetyloxy)-2-(β-D-glucopyranosyloxy)-3,16-dihydroxy-9-methyl-19-norlanosta-5,23-dien-22-one	12.993	C_38_H_60_O_11_	691.4063	691.4063	[M-H]^−^	0.00
Charantoside V/Karaviloside VII/Momordicoside G/Momordicoside F1	13.821	C_37_H_60_O_8_	677.4270	677.4273	[M+HCOO]^−^	0.44
Kuguaoside D/Kuguaoside B/Charantagenin D/Charantoside A/Momordicoside U/Goyaglycoside b/Momordicoside K	13.821	C_37_H_60_O_9_	693.4219	693.4211	[M+HCOO]^−^	−1.15
Kuguasaponin E/Kuguasaponin D/Kuguasaponin C/Kuguasaponin B/Goyaglycoside d/Goyaglycoside c	14.593	C_38_H_62_O_9_	661.4321	661.4328	[M-H]^−^	1.06

^a^ Deviation of measured *m*/*z* from calculated *m*/*z* values for a pseudomolecular ion generated from the molecular formula.

**Table 2:** Interaction profiles of compounds. Summary of docked compounds, their binding interactions at the active site, and the corresponding interacting residues.

Compounds	Interactions	Residues
**α-tocopherol**	Hydrophobic	VAL-26, TYR-31, VAL-34, ALA-36, LYS-49, THR-102, LEU-104, PHE-165, LEU-167, ALA-168
	Hydrogen bonding	PRO-25, ALA-168
**Ascorbic acid**	Hydrogen bonding	ALA-47, LYS-49, VAL-101, THR-102
**Quercetin**	Hydrogen bonding	MET-105, GLY-106, GLY-166, ALA-168, HIS-170

## Data Availability

The original contributions presented in this study are included in the article/Supplementary Materials. Further inquiries can be directed to the corresponding authors.

## Supplementary Materials

The following supporting information can be downloaded at https://www.mdpi.com/article/10.3390/ph19060893/s1: Figure S1: Effects of MCE on cell viability and nitric oxide production.

## Abbreviations

The following abbreviations are used in this manuscript:

TNF-α	Tumor Necrosis Factor-α
IL-6	InterLeukin-6
GO	Gene Ontology
KEGG	Kyoto Encyclopedia of Genes and Genomes
BP	Biological Process
MCE	*Momordica charantia* Extract
DEX	Dexamethasone
MAFbX	Muscle Atrophy F-box
MMP-2	Matrix Metalloproteinase-2
MMP-9	Matrix Metalloproteinase-9
MyoD	Myogenic Differentiation 1
p38 MAPK	p38 Mitogen-Activated Protein Kinase
p-p38	Phosphorylated p38
RMSD	Root Mean Square Deviation
PLIP	Protein–Ligand Interaction Profiler
AKT1	AKT Serine/Threonine Kinase 1
AMPK	AMP-Activated Protein Kinase
FOXO	Forkhead Box O
INS	Insulin
INSR	Insulin Receptor
IRS1	Insulin Receptor Substrate 1
NOS3	Nitric Oxide Synthase 3
