# Supplementary material for: Protective Effects of Momordica charantia Extract on Dexamethasone-Induced Sarcopenic Changes in C2C12 Cells: Integrated Network Pharmacology and Experimental Validation
Source: Pharmaceuticals (Basel). 2026 Jun 4;19(6):893. doi: 10.3390/ph19060893 (PMC13304634; doi:10.3390/ph19060893)
Supplement: Supplementary file 1 [file pharmaceuticals-19-00893-s001.zip › pharmaceuticals-4285321-supplementary.pdf]

## Supplementary Materials

# Protective Effects of *Momordica charantia* Extract on Dexamethasone-Induced Sarcopenic Changes in C2C12 Cells: Integrat-ed Network Pharmacology and Experimental Validation

**Jung Eun Park [1,†], Kang Sub Kim [1,†], Mina Jeong [1], Hee Woon Ann [1], Rajath Ramachandran [1], Il-Ho Park [2], Ki Hyun Kim[3], Ki Sung Kang [1,*] and Dae-Woon Eom [4,*]**

[1] College of Korean Medicine, Gachon University, 1342 Seongnamdae-ro, Sujeong-gu, Seongnam 13120, Republic of Korea; ppp1416@gachon.ac.kr (J.E.P.), kagnsub@gachon.ac.kr (K.S.K.), jmina@gachon.ac.kr (M.J.), heewoon05@naver.com (H.W.A.), rajath@gachon.ac.kr (R.R.)

[2] College of Pharmacy, Sahmyook University, Seoul 01795, Republic of Korea; parkilho@syu.ac.kr (I.-H.P.)

[3] School of Pharmacy, Sungkyunkwan University, 2066 Seobu-ro, Jangan-gu, Suwon 16419, Republic of Korea

[4] Department of Pathology, University of Ulsan College of Medicine, Gangneung Asan Hospital, Gangneung, 210-711, Republic of Korea; edwjyh@hanmail.net (D.-W.E.)

***** Correspondence: kkang@gachon.ac.kr; Tel.: +82-31-750-5402 (K.S.K.), edwjyh@hanmail.net; Tel.: +82-33-610-3428 (D.W.E.)

† These authors contributed equally to this study.

## Supporting Information Contents:

**Figure S1. Effects of MCE and quercetin on cell viability and nitric oxide production. S3**

## Supplementary Materials and Methods

### Nitric Oxide Production Assay

Nitric oxide (NO) production was measured using the Griess reaction. Briefly, RAW 264.7 cells were treated with MCE at the indicated concentrations in the presence or absence of lipopolysaccharide (LPS). After treatment, the culture supernatant was collected and mixed with an equal volume of Griess reagent. The mixture was incubated at room temperature, and the absorbance was measured at 540 nm using a microplate reader. NO production was calculated using a sodium nitrite standard curve and expressed as a percentage of the LPS-treated control group.

### Supplementary Results

Cell viability and NO production were evaluated to further assess the biological effects of MCE and quercetin. As shown in Figure S1A, treatment with MCE did not markedly reduce cell viability at the tested concentrations. In addition, NO production was measured to determine the effect of MCE on inflammatory mediator production. As shown in Figure S1B, MCE and quercetin modulated NO production without causing notable cytotoxicity. These results suggest that the observed biological effects were not primarily associated with cytotoxicity.

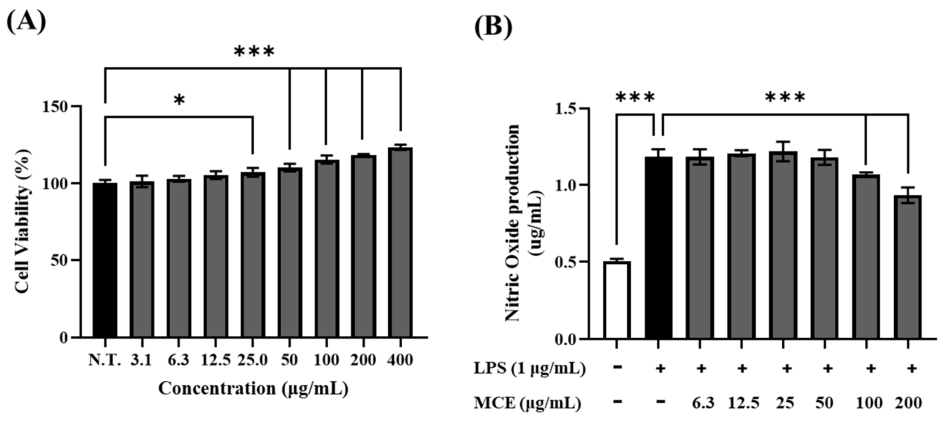


**Figure S1. Effects of MCE on cell viability and nitric oxide production.** (A) Cell viability was assessed after treatment with Momordica charantia extract or quercetin at the indicated concentrations. (B) NO production was measured using the Griess reaction in LPS-stimulated cells after treatment with MCE. Values are presented as the mean ± SD.
